# High-quality RNA extraction from the sea urchin *Paracentrotus lividus* embryos

**DOI:** 10.1371/journal.pone.0172171

**Published:** 2017-02-15

**Authors:** Nadia Ruocco, Susan Costantini, Valerio Zupo, Giovanna Romano, Adrianna Ianora, Angelo Fontana, Maria Costantini

**Affiliations:** 1 Department of Biology and Evolution of Marine Organisms, Stazione Zoologica Anton Dohrn, Villa Comunale, Napoli, Italy; 2 Department of Biology, University of Naples Federico II, Complesso Universitario di Monte Sant’Angelo, Via Cinthia, Napoli, Italy; 3 Bio-Organic Chemistry Unit, Institute of Biomolecular Chemistry-CNR, Via Campi Flegrei 34, Pozzuoli, Naples, Italy; 4 CROM, Istituto Nazionale Tumori “Fondazione G. Pascale”, IRCCS, Napoli, Italy; 5 Center of Villa Dohrn Ischia-Benthic Ecology, Department of Integrative Marine Ecology, Stazione Zoologica Anton Dohrn, P.ta S. Pietro, Ischia, Naples, Italy; 6 Department of Integrative Marine Ecology, Stazione Zoologica Anton Dohrn, Villa Comunale, Napoli, Italy; US Geological Survey, UNITED STATES

## Abstract

The sea urchin *Paracentrotus lividus* (Lamarck, 1816) is a keystone herbivore in the Mediterranean Sea due to its ability to transform macroalgal-dominated communities into barren areas characterized by increased cover of bare substrates and encrusting coralline algae, reduced biodiversity and altered ecosystem functions. *P*. *lividus* is also an excellent animal model for toxicology, physiology and biology investigations having been used for more than a century as a model for embryological studies with synchronously developing embryos which are easy to manipulate and analyze for morphological aberrations. Despite its importance for the scientific community, the complete genome is still not fully annotated. To date, only a few molecular tools are available and a few Next Generation Sequencing (NGS) studies have been performed. Here we aimed at setting-up an RNA extraction method to obtain high quality and sufficient quantity of RNA for NGS from *P*. *lividus* embryos at the pluteus stage. We compared five different RNA extraction protocols from four different pools of plutei (500, 1000, 2500 and 5000 embryos): TRIzol®, and four widely-used Silica Membrane kits, GenElute™ Mammalian Total RNA Miniprep Kit, RNAqueous® Micro Kit, RNeasy® Micro Kit and Aurum™ Total RNA Mini Kit. The quantity of RNA isolated was evaluated using NanoDrop. The quality, considering the purity, was measured as A260/A280 and A260/230 ratios. The integrity was measured by RNA Integrity Number (RIN). Our results demonstrated that the most efficient procedures were GenElute, RNeasy and Aurum, producing a sufficient quantity of RNA for NGS. The Bioanalyzer profiles and RIN values revealed that the most efficient methods guaranteeing for RNA integrity were RNeasy and Aurum combined with an initial preservation in RNAlater. This research represents the first attempt to standardize a method for high-quality RNA extraction from sea urchin embryos at the pluteus stage, providing a new resource for this established model marine organism.

## Introduction

NGS technologies have found broad applications in functional genomic research, including gene expression profiling, genome annotation, small ncRNA discovery and profiling, determination of DNA sequences associated with epigenetic modifications of histones and DNA, and profiling DNA methylations [1–6].

“Omic” approaches are powerful instruments not only for genomic studies but also for many other research fields. For example starting from 2007, ecologists used this approach to address several important ecological questions, introducing the new discipline of “ecological genomics” and/or “molecular ecology” [7]. Advances in DNA technologies have had a very strong impact on molecular ecology studies, also providing new tools for understanding the response of organisms to environmental stress [8–10]. The study of stress response in animal species has represented a predominant subject in the research, as a consequence of global warming, ocean acidification and increased pollution [10]. Echinoids have been considered ideal models for monitoring marine environmental hazards [11], because they are often key herbivore species, having a major role in structuring and controlling macroalgal assemblages, thereby shaping the benthic seascape, and also playing an important role in coastal food webs throughout the world [12–16]. They have traditionally been used as model organisms to study reproduction and early cell differentiation, sperm-oocyte interactions and apoptosis [17–19]. These organisms have been also proposed as valuable bioindicators for detecting environmental perturbations [20–23].

Among the echinoderms, the sea urchin *Paracentrotus lividus* is considered a suitable organism to study the ecotoxicological responses to xenobiotics and the physiological reactions to physical stressors [24–29]. It has also been used to test the effect of marine natural toxins, such as diatom-derived secondary metabolites, which have also apoptotic and anti-cancer activity [30,31].

The species represents a useful test organism for several reasons: it is an important component of benthic communities in the Mediterranean Sea and Atlantic Ocean; extraction and maintenance of gametes are easy; the embryos grow rapidly and synchronously (pluteus stage is reached 48 hours post-fertilization) and embryos are transparent and suitable for microscope detection of sub-lethal effects of pollutants on development; they have a sufficiently long reproductive season (from October to May). Although *P*. *lividus* represents a well-established model organism, to date the complete sequence of its genome is still not available [19]. Thanks to NGS approaches, it would be possible to fill this genome gap by increasing the amount of molecular information on the sea urchin *P*. *lividus*. However, high-throughput NGS of genomes and transcriptomes requires high-quality, clean, and concentrated DNA/RNA.

This study compares five RNA extraction protocols using embryos of the sea urchin *P*. *lividus* to define the best method for obtaining high-quality RNA for NGS applications. We fertilized four pools of eggs and performed RNA extractions from embryos at the pluteus stage (at 48 hour post-fertilization) preserved in RNA*later*^®^, using five different protocols: a guanidinium-thiocyanate-phenol-chloroform (GTPC) extraction protocol with TRIzol^®^, and four widely-used Silica Membrane kits, namely GenElute^™^ Mammalian Total RNA Miniprep Kit (Sigma-Aldrich), RNAqueous^®^ Micro Kit (Ambion from Life Technologies), RNeasy^®^ Micro Kit (Qiagen) andAurum^™^ Total RNA Mini Kit (Biorad). The quantity and quality of isolated RNA was evaluated taking into account the purity, measured as A260/A280 and A260/230 ratios, and the integrity, measured as RNA Integrity Number (RIN). The results yielded a first protocol for high-quality RNA extraction from sea urchin plutei, increasing the resources for such a well-established model organism.

## Materials and methods

### Ethics statement

*P*. *lividus* (Lamarck) were collected from a site in the Bay of Naples that is not privately-owned or protected in any way, according to Italian legislation (DPR 1639/68, 09/19/1980 confirmed on 01/10/2000). Field studies did not include endangered or protected species. All experimental procedures on animals were in compliance with the guidelines of the European Union (Directive 609/86).

### Sample collection and preservation

Adult sea urchins were collected during the breeding season by scuba-diving in the Gulf of Naples, transported in thermic box to the laboratory within 1 hour after collection and maintained in tanks with circulating sea water until testing. Sea urchins were injected with 2 M KCl through the peribuccal membrane to obtain the emission of gametes. Eggs were washed with filtered sea water (FSW) and kept in FSW until use. Concentrated spermatozoans were collected, dried and kept undiluted at +4°C until use.

Different quantities of eggs (500, 1000, 2500 and 5000) from five females were fertilized, using sperm-to-egg ratios of 100:1, and embryos were then collected at the pluteus stage (48 hpf) by centrifugation at 1800 rcf for 10 min in a swing out rotor at 4°C. Immediately after harvesting, the embryos were placed in at least 10 volumes of the RNA*later*^®^, an RNA Stabilization Reagent (Qiagen, Hilden, Germany), and then frozen in liquid nitrogen and kept at -80°C.

### RNA extraction

Five different methods of RNA extraction were compared.

i) TRIzol^®^ RNA extraction method

Total RNA was extracted using TRIzol (Invitrogen, Life Technologies, Carlsbad, CA, USA) according to the manufacturer’s instructions, homogenizing with TissueLyser (Qiagen, Austin, TX, US) with 3 mm sterile aluminium beads at 20.1 Hertz (Hz) for 5 minutes. Extractions with chloroform/isoamyl alcohol (24:1) were performed, followed then by RNA precipitation by adding glycogen and isopropyl alcohol. Total RNA was suspended in 0.1% v/v diethylpyrocarbonate (DEPC)-treated water. Contaminating DNA was degraded by treating each sample with a DNase RNase-free kit (Roche, Milan, Italy) according to the manufacturer’s instructions. The samples were then stored at -80°C.

ii) GenElute™ Mammalian Total RNA Miniprep Kit

Embryo lysis was performed in 500 μl Lysis Solution/2-ME mixture and then RNA was extracted following the manufacturer's protocol. An additional step with DNase RNase-free kit (Roche, Milan, Italy) was used to remove contaminating DNA. Finally, RNA was eluted with 50 μl Elution Solution provided by the manufacturer. The samples were then stored at -80°C.

iii) RNeasy^®^ Micro Kit

Embryos were lysed with 350 μl Buffer RLT/2-ME (10 μl β-mercaptoethanol for each ml of Buffer RLT) and homogenized with TissueLyser (Qiagen, Austin, TX, US) using 3 mm sterile aluminium beads at 20.1 Hz for 5 minutes. RNA was extracted following the manufacturer's protocol. DNA contaminations were avoided using the RNase-Free DNase Set, provided by the kit. RNA was eluted with 14 μL RNase-free water. The samples were then stored at -80°C.

iv) RNAqueous^®^ Micro Kit

Pellets were suspended in 500 μl Lysis Solution by vortexing vigorously. Elution was performed in two steps, adding 10 μl Elution Solution each time, and preheating at 75°C. Finally a DNase treatment was performed adding 1/10 volume 10X DNase I Buffer and 1 μL of DNase I to the sample. DNase was finally blocked using a DNase Inactivation Reagent (1/10 of total volume). The samples were then stored at -80°C.

v) Aurum^™^ Total RNA Mini Kit

Samples were disrupted with 350 μl Lysis Solution (already supplemented with 1% β-mercapto-ethanol), pipetting up and down several times to lyse cells thoroughly. After treatment with 80 μl of diluted DNase I (75 μl DNase Dilution Solution and 5 μl DNase I), RNA was eluted with 40 μl of Elution Solution. The samples were then stored at -80°C.

### Determination of RNA quantity and quality

The amount of total RNA extracted with the five methods was estimated by measuring the absorbance at 260 nm and the purity at 260/280 and 260/230 nm ratios, by a Nanodrop (ND- 1000 UV-Vis Spectrophotometer; NanoDrop Technologies, to exclude the presence of proteins, phenol and other contaminants [32]. The integrity measurements of RNA were finally assessed by running 100–200 ng of RNA samples in each line of a 6000 Nano LabChip in an Agilent Bioanalyzer 2100 Bioanalyzer (Agilent Technologies, Santa Clara, CA, US). RNA integrity was measured using the RIN value, which was calculated based on the comparison of the areas of 18S rRNA and 28S rRNA [33]. RIN values over a threshold of 8 were considered to indicate non-degraded RNA extraction methods.

### Statistical analysis

The total number of sample extractions from four different amounts of embryos is reported in Table 1. RNA quantity and quality (A260/280 and A260/230 ratios, and RIN values) using different RNA extraction procedures were tested using One-way Variance Analysis (ANOVA). We first checked whether measures of RNA quality, absorbance ratios, A260/230 and A260/280, and RIN values were correlated in our data by a nonparametric Spearman’s correlation coefficient. Moreover, we verified that their differences were statistically different using the t-test using whereby p-values lower than 0.05 were considered significant. Distribution of RIN values was graphically represented by boxplots for different treatments of sea urchin embryos. Statistical analyses were performed using GraphPad PRISM v.4 software (San Diego, CA, US).

**Table 1:** Extraction number, total RNA quantity (μg), purity (A260/280 and A260/230) and integrity (RIN values) from different number of *P*. *lividus* embryos using five different extraction methods: TRIzol, GenElute™ Mammalian Total RNA Miniprep Kit (Sigma-Aldrich), RNAqueous® Micro Kit (Ambion from Life Technologies), RNeasy® Micro Kit (Qiagen) and Aurum™ Total RNA Mini Kit (Biorad). Values represent mean ± SD. Values in bold in the RIN column are the average values among the RIN values obtained at the different number of *P*. *lividus* embryos. N/A indicates samples in which no values are assigned.

Extraction method	Extraction number	Embryos	RNA quantity (μg)	A260/230	A260/280	RIN
* *	* *	** **				
***TRIzol***	4	500	2.4 ± 0.23	1.96 ± 0.44	1.81 ± 0.24	N/A
* *	7	1000	3.5 ± 0.08	1.68 ± 0.18	1.42 ± 0.37	4.20 ±1.13
* *	7	2500	8.1 ± 0.16	1.85 ± 0.51	1.91 ± 0.42	4.60 ± 0.89
* *	7	5000	8.9 ± 0.09	1.83 ± 0.67	1.86 ± 0.30	3.90 ± 0.67
* *						**4.20**
* *	* *					
***GenElute***	4	500	3.2 ± 0.56	0.86 ± 0.87	2.00 ± 0.03	8.10 ± 0.03
* *	7	1000	4.2 ± 2.05	1.21 ± 0.05	1.98 ± 0.05	7.80 ± 0.05
* *	7	2500	12.8 ± 8.16	1.83 ± 0.03	1.93 ± 0.03	7.95 ± 0.35
* *	7	5000	25.9 ± 17.96	1.97 ± 0.03	1.96 ± 0.03	7.95 ± 0.07
* *	* *					**7.95**
* *	* *					
***RNeasy***	4	500	1.0 ± 0.27	0.50 ± 0.41	2.15 ± 0.25	9.20 ± 0.01
* *	7	1000	1.8 ± 0.14	0.78 ± 0.61	1.92 ± 0.26	9.45 ± 0.49
* *	7	2500	8.9 ± 0.05	2.35 ± 0.07	1.97 ± 0.01	9.30 ± 0.14
	7	5000	18.7 ± 7.91	2.17 ± 0.08	1.98 ± 0.05	9.20 ± 0.15
* *						**9.30**
* *	* *					
***RNAqueous***	4	500	1.6 ± 2.1	0.46 ± 0.71	1.90 ± 0.34	7.90 ± 0.08
* *	7	1000	2.9 ± 3.6	0.72 ± 1.07	1.88 ± 0.48	8.10 ± 0.27
* *	7	2500	3.0 ± 3.9	2.37 ± 0.29	1.83 ± 0.28	10.0 ± 0.17
	7	5000	4.2 ± 5.3	2.35 ± 0.17	1.93 ± 0.14	8.90 ± 0.09
						**8.70**
* *	* *					
***Aurum***	4	500	1.6 ± 0.71	1.96 ± 1.14	2.04 ± 0.25	9.65 ± 0.50
* *	7	1000	5.4 ±1.90	1.93 ± 0.36	2.13 ± 0.08	9.45 ± 0.35
* *	7	2500	13.3 ± 2.49	1.65 ± 1.18	2.08 ± 0.07	9.95 ± 0.07
	7	5000	23.0 ± 1.47	1.94 ± 0.75	2.05 ± 0.07	9.85 ± 0.07
						**9.70**

## Results

Increasing amounts of sea urchin *P*. *lividus* eggs (500, 1000, 2500 and 5000) were fertilized and embryonic development was followed until the pluteus stage, which is reached at about 48 hpf. The results of different RNA extractions are summarized in Table 1. Concerning RNA quantity, similar results were obtained using TRIzol, RNeasy and RNAqueous methods for 500 and 1000 embryos. In the case of 2500 and 5000 embryos, significantly higher quantity of RNA was extracted using the GenElute, RNeasy and Aurum methods (One-way ANOVA p<0.0001), whereas no significant differences were detected between 2500 and 5000 embryos versus 500 and 1000 embryos with the RNAqueous method (p>0.05). All extraction procedures from 2500 and 5000 embryos yielded sufficient amounts of total RNA as requested for NGS approaches (~2–3 μg of total RNA). We did not detect significant differences in RNA purity (A260/230 and A260/280 ratios) using the five considered methods. The Spearman’s coefficients did not reveal a correlation between RNA quality variables based on absorbance ratios (A260/280 and A260/230) and RIN values (ρ ranged from 0.40 to 0.52; p values from 0.0833 to 1.0; for further details see S1 Table). These results were also confirmed by t-test, showing that RNA quality and RIN trends were statistically different (p < 0.0001).

**Fig 1:**
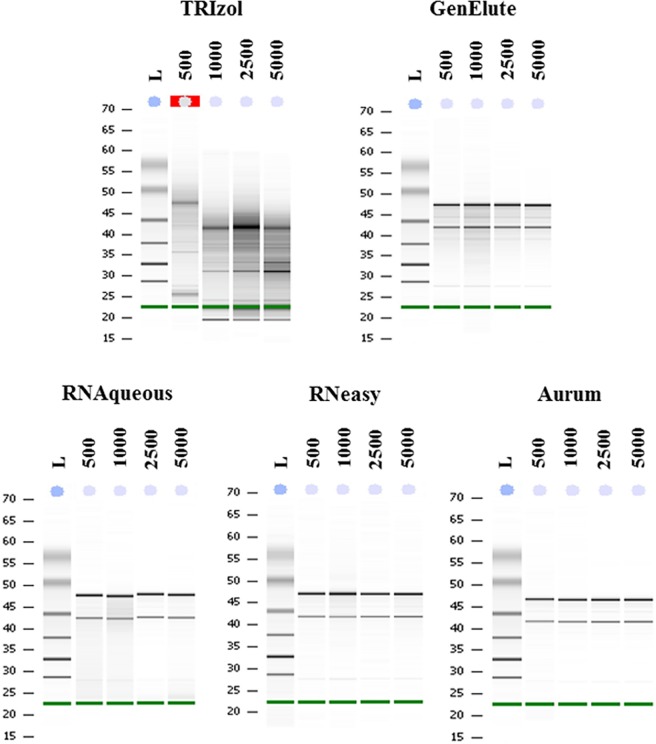
Bioanalyzer Agilent electrophoresis runs. Examples of representative Bioanalyzer Agilent electrophoresis runs for the five different methods applied for RNA extractions from *P*. *lividus* embryos: TRIzol, GenElute™ Mammalian Total RNA Miniprep Kit (Sigma-Aldrich), RNAqueous® Micro Kit (Ambion from Life Technologies), RNeasy® Micro Kit (Qiagen) and Aurum™ Total RNA Mini Kit (Biorad). Four different numerical amounts of embryos were used for RNA extraction: 500, 1000, 2500 and 5000 embryos. The ladder (L) is reported in the first lane of each run. The green band at the bottom of each panel is the RNA 6000 Nano Marker (Agilent RNA 6000 Nano Kit, Agilent Technologies, Inc.). Red box in the 500 lane of TRIzol indicates that the Bioanalyzer software cannot calculate RIN values (reported as N/A in the Table 1) for this sample, because of very low concentration and high level of degradation of the RNA.

Representative Bioanalyzer Agilent electrophoresis runs showed low or high quality of total RNA extracted from *P*. *lividus* embryos (Fig 1).

**Fig 2:**
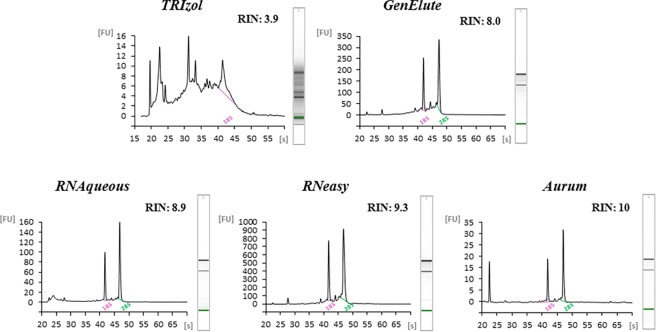
Agilent Bioanlyzer electropherograms. Examples of representative Agilent Bioanlyzer electropherograms of *P*. *lividus* RNA: for TRIzol, GenElute and RNAqueous RNA extraction from 5000 embryos extraction; for RNeasy and Aurum RNA extraction from 2500 embryos (see also Table 1). Relative Fluorescent Unit (FU) and seconds of migration (s) of RNA samples isolated according to the five different extraction methods are reported. RIN values are also reported.

The methods used for total RNA extractions consistently showed two bands, corresponding to 28S and 18S rRNA, with the only exception being the TRIzol method, that showed degraded RNA with several bands. These results were also confirmed by the representative Bioanalyzer Agilent profiles showing electropherograms with low or high quality of total RNA extracted from different numerical amounts of *P*. *lividus* embryos along with corresponding RIN values (Fig 2).

Total RNA extracted with TRIzol showed a single peak corresponding to 18S at 42 seconds (s), absence of a 28S peak, a high amount of small size RNA occurring between 25s and 42 s and RNA degradation with a very low RIN value (RIN = 3.90). The integrity of *P*. *lividus* plutei RNA significantly improved with the use of Silica Membrane methods. In fact, in all four commercial kits applied for RNA extraction, the electropherograms showed two peaks corresponding to 28S (42s) and 18S (47s) with very high average RIN values ranging from 7.9 for GenElute, 8.7 for RNAqueous, increasing to 9.3 for RNeasy and reaching a maximum value of 9.7 with the Aurum kit. Such high RIN values were considered suitable for NGS technologies.

In samples exhibiting A260/280 and A26030 ratios lower than 1.8 or over 2.0 (Table 1), we observed RIN values higher than 8. These samples also showed intact 28S and 18S. The distributions of RIN were also reported in the boxplot of RIN values (S1 Fig). Extraction by RNAqueous was the most unstable method based on RIN values (from 7.90 to 10.0). Moreover, even if GenElute and Aurum methods yielded comparable results producing higher quantities of RNA (Table 1), the boxplot clearly showed that they were not comparable in terms of RNA integrity. In fact, the GenElute method produced RNA with RIN values lower than those obtained with the Aurum method.

## Discussion

In the present work we focused our attention on optimizing RNA extraction protocols from sea urchin *P*. *lividus* embryos at the pluteus stage. Since the sea urchin *P*. *lividus* is one of the most common sea urchins in the Mediterranean Sea, as well as in the Northeast Atlantic from Ireland to the coasts of Morocco, the Canary Islands and the Azores [34], setting up a feasible method of RNA extraction represents the first step to afford details of its trophic and reproductive biology. *P*. *lividus* is considered a keystone herbivore, able to transform communities dominated by macroalgae into barren areas thereby reducing biodiversity and altering ecosystem functions [35,36]. The sea urchin has been extensively used as an invertebrate model organism in developmental biology and in ecotoxicology studies to assess the effects of marine pollution on marine organisms [19].

Despite the well-recognized ecological significance of the sea urchin in marine environment, to date there are few molecular tools available for this species. For this reason, NGS approaches are increasingly being applied to this species, in order to increase the molecular data on *P*. *lividus*. To our knowledge, our work represents the first attempt to standardize a method for RNA extraction from sea urchin embryos at the pluteus stage. Our aim was to obtain high-quality RNA in sufficient quantities for NGS technologies. Firstly, we extracted RNA from different numbers of sea urchin *P*. *lividus* embryos at the pluteus stage, applying five different protocols. Our findings revealed that all extraction procedures from 500 and 1000 embryos did not yield sufficient amounts of total RNA as requested for NGS approaches (~2–3 μg of total RNA). Sufficient RNA quantity was obtained from 2500 and 5000 embryos. The most efficient procedures to produce such quantities were obtained with GenElute, RNeasy and Aurum, producing more than 10 and 20 μg of RNA, using 2500 and 5000 embryos, respectively.

Having established the number of embryos necessary to obtain the required quantity of RNA, we assessed RNA quality by measuring different features: A260/280 and A260/230 ratios and estimation of RIN values [32,37–39]. In our samples, the most efficient strategies for assessing RNA integrity was represented by the estimation of the RIN value using Agilent Bioanalyzer chips. The Bioanalyzer profiles showed that GenElute, RNeasy and Aurum procedures were only comparable for RNA quantity, not for RNA integrity. In fact, RIN values using the GenElute procedure were the lowest in comparison to the other Silica Membrane procedures applied. RNAqueous was the most variable method whereas the most efficient for RNA integrity were RNeasy and Aurum.

Interestingly our findings demonstrated no significant correlations between A260/280 and A260/230 ratios and RNA integrity. A possible explanation for this may be due to the different stability of the RNAs. In fact, ribosomal RNA is more stable than mRNA [32, 40–43]. Moreover, the A260/280 ratio could have values of about two, because of the intact ribosomal RNA, even if the mRNA was degraded. This is, for example, the case of RNA samples after TRIzol extraction, suggesting that the method of preservation of our embryo samples represents a very important step to obtain high-quality RNA. Preservation in RNA*later* ensured greater stability for our samples since this a nontoxic storage reagent able to permeate cells and/or tissues and to stabilize and protect cellular RNA. This step minimizes the need for immediate processing of the samples, without jeopardizing the quality or quantity of RNA obtained after subsequent RNA isolation. RNA extraction from fresh cell/tissues has been used in many cases with success [23,41,43], but several laboratory conditions or field experiments do not allow for direct extraction upon collection. There are several indications that RNA*later* is a reliable preservative for RNA in a wide array of tissues improving the yield of total RNA [32,39].

This study fills a gap considering that no comparative studies for RNA extractions are available in the literature for sea urchin *P*. *lividus* plutei. A similar methodological analysis was performed by Pérez-Portela and Riesgo [43], although they did not use embryos at the pluteus stage (as in our case) from *P*. *lividus*, but tissues from another sea urchin, *Arbacia lixula*. In fact, their study aimed to optimize a preservation protocol for the isolation of high-quality RNA from three different *A*. *lixula* tissues: gonad, oesophagus and coelomocytes. Extractions of total RNA were performed with a modified TRIzol protocol for all tissues, applying four preservation treatments. The results showed high values of RNA quantity and quality for all tissues, indicating non-significant differences among samples. Insufficient RNA amount and great variability in RNA integrity were found in coelomocytes in RNA*later*. The most efficient system to stabilize RNA was the TRIzol method that produced high RNA quality and quantity. These data are comparable with our results (see above), because no correlation was found between RNA integrity and absorbance ratios, as in our experiments. Furthermore, the evaluation of RIN values by Agilent Bioanalyzer chips was the best approach to evaluate the RNA integrity (as in our case).

The extraction of high-quality RNA represents a fundamental step, considering that transcriptomic information and NGS approaches represent significant tools to acquire information regarding several biological processes. Several attempts, mainly for non-well-established model organisms, were recently made to establish methods for high-quality RNA extractions, because NGS represents the only way to address specific ecological and evolutionary questions [42,44].

Asai et al. [44] compared three commonly-used methods: TRIzol^®^, Aurum Total RNA Mini Kit and Qiagen RNeasy Micro Kit, in combination with preservation reagents TRIzol^®^ or RNA*late*r^®^, to obtain high-quality and large quantities of RNA from the copepod *Calanus helgolandicus*. Their results confirmed that the preservation of copepods in RNA*late*r^®^ and the extraction with Qiagen RNeasy Micro Kit were the optimal isolation method for high-quality and quantity of RNA for NGS studies of *C*. *helgolandicus*.

In conclusion, our results provide for the first time a standard protocol for isolating high-quality RNA from sea urchin plutei for high-throughput NGS studies thereby increasing the resources available for a well-established model organism such as *P*. *lividus*.

## Supporting information

S1 Fig Boxplot of RIN values obtained with the five different methods of RNA extraction. The boxes extend from the 25th to the 75th percentile, and the line in the middle is the median. The error bars extend down to the lowest value and up to the highest. Dashed line at the RIN value of 8 is reported, because higher values than 8 are considered suitable for NGS analysis.
(PPT)

S1 Table Correlation between RNA quality based on absorbance ratios (A260/280 and A260/230) and RIN values by Spearman's correlation coefficient and evaluation of their statistical difference by t-test, using the five different RNA extraction protocols for P. lividus embryos. We concern as statistically significant the p-values lower than 0.05.
(DOC)
