# Supplementary figures and images for: High-quality RNA extraction from the sea urchin Paracentrotus lividus embryos
Source: PLoS One. 2017 Feb 15;12(2):e0172171. doi: 10.1371/journal.pone.0172171 (PMC5310894; doi:10.1371/journal.pone.0172171)

## Slide 1
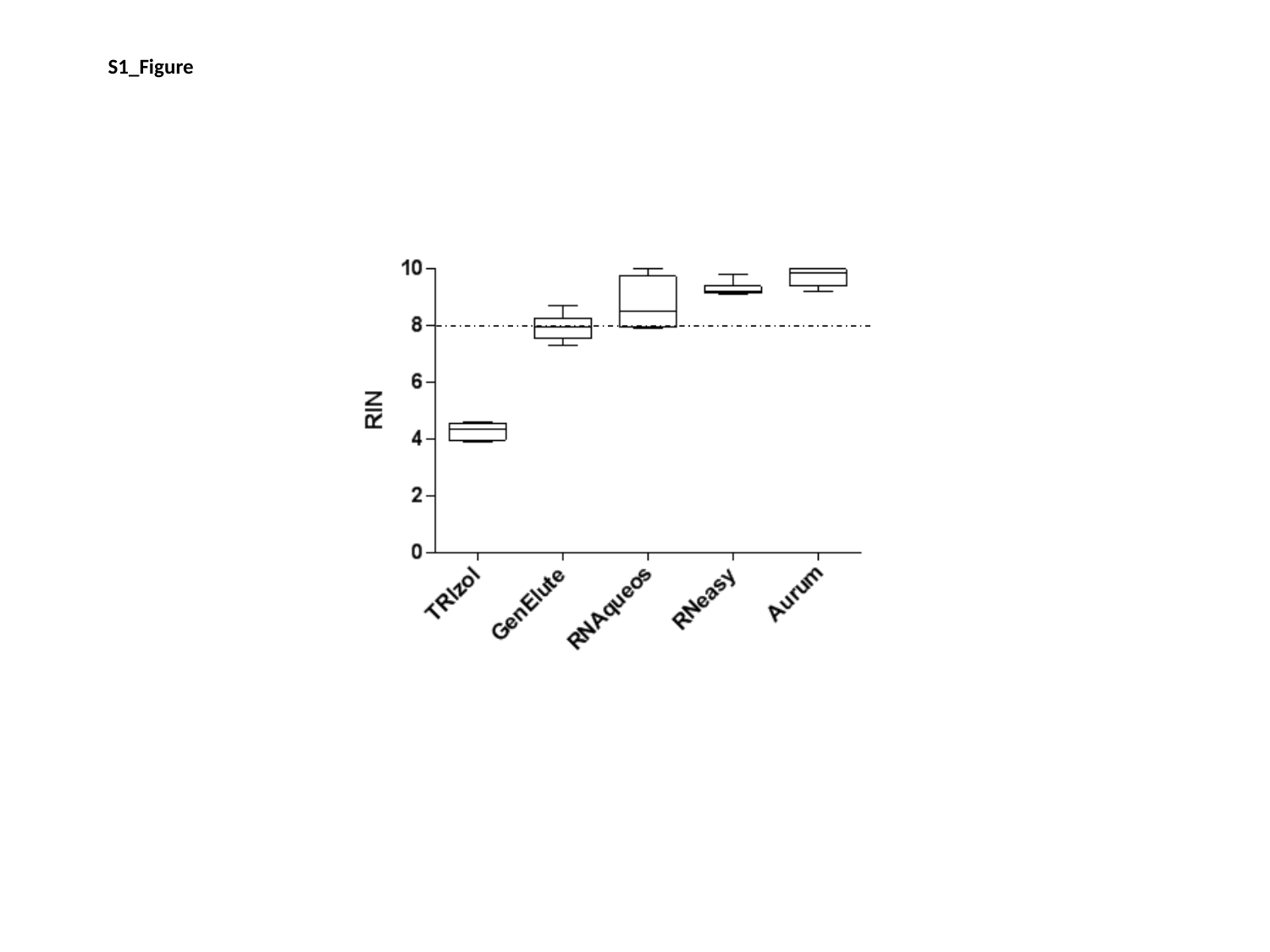

S1_Figure

Supplement: S1 Fig — The boxes extend from the 25th to the 75th percentile, and the line in the middle is the median. The error bars extend down to the lowest value and up to the highest. Dashed line at the RIN value of 8 is reported, because higher values than 8 are considered suitable for NGS analysis. (PPT) [file pone.0172171.s001.ppt]
