# Supplementary material for: High-quality RNA extraction from the sea urchin Paracentrotus lividus embryos
Source: PLoS One. 2017 Feb 15;12(2):e0172171. doi: 10.1371/journal.pone.0172171 (PMC5310894; doi:10.1371/journal.pone.0172171)
Supplement: S1 Table — We concern as statistically significant the p-values lower than 0.05. (DOC) [file pone.0172171.s002.doc]

**Supplementary Table S1.** Correlation between RNA quality based on absorbance ratios (A260/280 and A260/230) and RIN values by Spearman's correlation coefficient and evaluation of their statistical difference by t-test, using the five different RNA extraction protocols for P. lividus embryos. We concern as statistically significant the p-values lower than 0.05
